# Sex-Differences and Associations Between Complement Activation and Synovial Vascularization in Patients with Late-Stage Knee Osteoarthritis

**DOI:** 10.3389/fimmu.2022.890094

**Published:** 2022-05-24

**Authors:** Emily U. Sodhi, Holly T. Philpott, McKenzie M. Carter, Trevor B. Birmingham, C. Thomas Appleton

**Affiliations:** ^1^ Department of Physiology & Pharmacology, Schulich School of Medicine and Dentistry, Western University, London, ON, Canada; ^2^ Health & Rehabilitation Sciences, Faculty of Health Sciences, Western University, London, ON, Canada; ^3^ Bone & Joint Institute, Western University, London, ON, Canada; ^4^ Department of Medicine, Schulich School of Medicine, Western University, London, ON, Canada

**Keywords:** complement, synovium, osteoarthritis, sex-differences, inflammation, histopathology, vascularization

## Abstract

**Purpose:**

Synovial inflammation in knee osteoarthritis (OA) causes disorganized synovial angiogenesis and complement activation in synovial fluid, but links between complement and synovial microvascular pathology have not been established. Since complement causes vascular pathology in other diseases and since sex-differences exist in complement activation and in OA, we investigated sex differences in synovial fluid complement factors, synovial tissue vascular pathology, and associations between complement and synovial vascular pathology in patients with late-stage knee OA.

**Methods:**

Patients with symptomatic, late-stage radiographic knee OA undergoing total knee arthroplasty or high tibial osteotomy provided matched synovial fluid and tissue biopsies during surgery. Complement factors (C2, C5, adipsin, MBL, and CFI) and terminal complement complex (sC5b-C9) were measured in synovial fluid by multiplex or enzyme-linked immunosorbent assay, respectively. Features of synovial vascular pathology (vascularization, perivascular edema, and vasculopathy) were assessed by histopathology. Multivariate linear regression models were used to assess associations between synovial fluid complement factors and histopathological features of vascular pathology, with adjustment for age, sex, body mass index, and sex interaction. Sex-disaggregated comparisons were completed.

**Results:**

Synovial fluid biomarker and histopathology data were included from 97 patients. Most synovial fluid complement factors and synovial tissue histopathological features were similar between sexes. Synovial fluid C5 trended to lower levels in males (-20.93 ng/mL [95%CI -42.08, 0.23] *p=*0.05). Median vasculopathy scores (0.42 [95%CI 0.07, 0.77] *p=*0.02) were higher in males. In the full cohort, C5 concentration was associated with lower vascularization scores (-0.005 [95%CI -0.010, -0.0001] *p=*0.04) while accounting for sex*C5 interaction. In sex-disaggregated analyses, increased C5 concentration was associated with lower vascularization scores (-0.005 [95%CI –0.009, -0.0001] *p=*0.04) in male patients, but not in female patients. Males had higher sC5b-C9 compared to females. Additionally, males with high C5 had a higher synovial fluid concentration of sC5b-C9 compared to males with low C5. No differences were found in females.

**Conclusion:**

Higher synovial fluid C5 levels were associated with increased complement activation and decreased synovial vascularization in males but not in females with OA. Future studies should test whether synovial fluid complement activation suppresses synovial angiogenesis and identify mechanisms accounting for C5-related sex-differences in synovial fluid complement activation in patients with knee OA.

## 1 Introduction

Synovial inflammation in knee osteoarthritis (OA) causes disorganized synovial angiogenesis and complement activation in synovial fluid, but links between complement and synovial microvascular pathology have not been established. The formation of new blood vessels by angiogenesis is stimulated during wound healing and dysregulated by chronic inflammation (1). In knee OA, chronic synovial inflammation (synovitis) is associated with increased joint pain and disease progression (2). Synovitis disrupts the homeostatic roles of synovial tissue including synovial fluid production (3), nourishing articular cartilage (3), clearance of tissue turnover products (3), and joint tissue regeneration (4). Synovial vascular pathology in OA demonstrates angiogenesis (vascularization) (5), thick-walled microvessels (vasculopathy) (6), and perivascular edema (7). The mechanisms driving synovial angiogenesis in knee OA are not well understood (5, 8). Scanzello et al. proposed that synovitis in OA resembles a chronic wound, where disorganized angiogenesis may be a sign of attempted wound healing (9). We considered that complement may be associated with synovial vascular pathology in this study since complement-driven inflammation also disturbs wound healing, including regulation of angiogenic processes (10).

Inflammation in OA primarily involves components of the innate immune system, including activation of the complement cascade (11, 12). Complement activation occurs through three activation pathways (alternative, classical, and lectin), which ultimately converge in a terminal effector pathway (10). Downstream inflammatory effects of complement activation include increased blood vessel permeability (13), histamine release from mast cells (13), smooth muscle contraction (14), synthesis of angiogenic factors (15, 16), regulation of apoptosis, and release of chemoattractants by immune cells (13, 17), all of which may exacerbate synovial inflammation within the knee joint.

Wang et al. reported that the synovium and synovial fluid of knee OA patients have higher gene expression of complement effectors and lower complement inhibitors compared to healthy individuals (18). Knockout of common and alternative complement pathway components also protected against the development of structural joint damage in multiple rodent models of knee OA, but synovial vascular outcomes were not assessed. To our knowledge, there are presently no published studies investigating relationships between complement and synovial vascular pathology in knee OA.

Female sex is a major OA risk factor (19). Females are 1.4 times more likely than males to be diagnosed with OA after age 65 (20), suffer from more severe symptoms (21), and have lower responses to current therapies than males (22). Interestingly, sex-differences in complement activation have also been identified. For example, serum samples from healthy females demonstrated lower alternative pathway activation and lower common/terminal pathway and mannose-binding lectin components compared to males (23). Accordingly, it is recommended that the effects of sex be evaluated in studies investigating complement-associated pathologies. Considering the well-recognized sex-differences in both knee OA epidemiology and complement activation, our objectives were to investigate sex differences in complement factors in synovial fluid, synovial tissue vascular pathology, and associations between complement factors and vascular pathology in patients with late-stage knee OA.

## 2 Methods

### 2.1 Study Participants

Participants were recruited from the Western Ontario Registry for Early Osteoarthritis (WOREO) Knee Study, a prospective cohort designed to investigate clinical, biomechanical, and pathophysiological features of inflammation in patients with knee OA. The present study included 97 consecutively recruited patients with symptomatic, late-stage knee OA. Eligibility criteria included patients with Kellgren-Lawrence (KL) grades of 3 or 4 (24), frequent knee pain, and compromised function indicating surgical management by total knee arthroplasty or high tibial osteotomy. Patients without synovial histopathology and synovial fluid data were excluded. One patient reported currently taking hormone replacement therapy. A sample of n=85 was required to detect a Cohen’s *f^2^* of 0.15 (moderate effect size) with 80% power and an α = 0.05, indicating we were adequately powered for our primary analysis with the total cohort (n=97) (25). Knee radiographs were acquired at study enrollment. Participants provided written, informed consent and the study was approved by Western University’s Research Ethics Board for Health Sciences Research Involving Human Subjects (HSREB #109255). Demographic measures including age, sex, and body mass index (BMI) were collected. Patient-reported pain in the target (study) knee was measured using the Knee Osteoarthritis Outcome Score (KOOS) pain subscale, normalized to 100%, where 100 corresponds to no pain and 0 to the worst pain possible.

### 2.2 Synovial Fluid Collection and Complement Concentrations in Synovial Fluid

At the time of surgery, prior to knee joint arthrotomy, synovial fluid was aspirated to avoid contamination by blood. Synovial fluid samples were then centrifuged at 2800g and at 4°C for 15 minutes. Supernatants were aliquoted into cryovials for storage at -80°C until use. Synovial fluid samples with sufficient volume and minimal blood contamination were selected for multiplex analysis. Samples were treated with hyaluronidase (1 µg/mL) prior to assay with the Human Complement-2, Milliplex Multiplex Assay using Luminex (Millipore, USA), performed commercially at Eve Technologies, Calgary, AB, Canada, to measure the concentration of complement factors C5, C2, CFI, adipsin (factor D), and MBL (mannose-binding lectin) (ng/mL). We excluded the short-lived, unstable intermediates C4b and C5a in these *in vivo* samples as we did not use protease inhibitors at the time of synovial fluid processing and this assay does not measure the more stable glycosylated (des Arg) metabolite.

### 2.3 Synovial Fluid Concentrations of Terminal Complement Complex (sC5b-C9)

To assess levels of a stable complement activation product and ensure that lower C5 levels were not due to consumption due to rapid C5 cleavage/activation, synovial fluid samples from a subset of patients that had adequate synovial fluid volume were treated with hyaluronidase prior to measuring terminal complement complex (sC5b-C9) using an enzyme-linked immunosorbent assay (ELISA) kit according to manufacturer’s protocol (Svar Life Science, Malmö, Sweden). We included samples from 16 male and 16 females patients, with n = 8 per quartile (highest/lowest) of C5 concentration. Absorbance reading at 620 nm (reference) was subtracted from the absorbance reading at 450 nm. Amount of sC5b-C9 (ng/mL) present in each sample was interpolated using a standard curve.

### 2.4 Synovial Tissue Histopathology

To standardize the location of tissue collection, all synovial tissue biopsies were obtained from the lateral suprapatellar recess at the time of surgery. After overnight fixation in 4% paraformaldehyde, synovial tissue samples were processed, and paraffin embedded. Serial sections (5 µm thickness) were mounted on glass slides, stained with hematoxylin and eosin, and 5 high powered fields (hpf) per patient at least 100µm apart were assessed. Six histopathological features were graded as described previously (7): perivascular edema, fibrosis, sub-synovial infiltrate, surface fibrin deposition, synovial lining thickness, and vascularization. A seventh feature, vasculopathy, was graded according to the number of thick-walled vessels (lumen diameter is smaller than vessel wall thickness) per high powered field (hpf) and was adapted from Philosophe et al. (2014) (26). Our primary analysis focused on measures of vascular pathology. Vascularization and perivascular edema were graded 0-3 (none-severe) (7). Vasculopathy was graded 0-3 as follows: Grade 0 = no thick-walled vessels per hpf; Grade 1 = <1/3 thick-walled vessels per hpf; Grade 2 = 1/3-2/3 thick-walled vessels per hpf; Grade 3 = >2/3 thick-walled vessels per hpf. The mean and median grades of all hpf per patient were calculated for each histopathological feature.

### 2.5 Synovial Tissue Immunofluorescence

To assess classical pathway activation in synovial tissue, we performed immunofluorescent detection for C1q. Slides were heated at 62°C for 30 minutes, cooled to room temperature, and then deparaffinized and rehydrated. For antigen retrieval, sections were immersed in 70°C Tris buffer (10 mM Tris, 1 mM EDTA, 10% glycerol pH 9) and allowed to cool to below 30°C. Sections were permeabilized by incubation in 0.2% Triton-X in 1x PBS for 10 minutes. Slides were rinsed with 1X phosphate buffered saline (PBS) and blocked with 5% bovine serum albumin blocking solution (1x PBS, 5.0% BSA, 0.1% Triton-X). Samples were incubated overnight at 4°C with anti-human C1q (1:100; ab268120; Abcam, Cambridge, UK). We included negative (no primary antibody) control sections. Goat anti-mouse conjugated to Alexa Fluor-647 (1:500; Jackson ImmunoResearch Laboratories) in 1X PBS was added to the slides for an hour at room temperature. Slides were mounted with Molecular Probes Prolong Gold Antifade Mountant with DAPI (Fisher Scientific, P36931).

Slides were imaged at 40X using a Leica Aperio VERSA 8 microscope scanner located at Molecular Pathology Core Facility, Robart’s Research Institute. Immunofluorescence images were analyzed quantitatively using QuPath (v0.3.1) (27) pixel classifier tools to measure percent positive cells for C1q.

### 2.6 Statistical Analyses

All analyses were done using *Graphpad Prism 8* (GraphPad Software, San Diego, California USA) or Stata IC/15.1 (StataCorp LLC, College Station, TX, USA).

#### 2.6.1 Descriptive Statistics

Descriptive statistics for patient’s demographics and clinical characteristics were calculated as mean +/- standard deviation (range) and frequency (percentage of total) for continuous and categorical variables, respectively. Two-tailed independent student’s t-tests were used to compare age, BMI, and KOOS pain between sexes. A Fisher’s Exact test was used to compare proportions of KL grades between sexes. Statistical significance was set at p<0.05.

#### 2.6.2 Sex-Based Analyses

Unpaired, two-tailed t-tests (or Mann-Whitney U tests in case of non-normally distributed data as assessed using QQ plots) were conducted to assess differences in synovial fluid complement concentrations between sexes. Measures were reported as the difference in mean concentrations +/- 95% confidence intervals. To assess for relationships between synovial fluid levels of C5 and other measured complement components, we fitted a series of sex-disaggregated multivariate linear regression models with adjustment for age and BMI. Results were reported as unstandardized beta coefficients +/- 95% confidence intervals.

To assess sex-differences in complement activation, we used an unpaired, two-tailed t-test to test if terminal complement complex (sC5b-C9) was different between males and females. To determine if C5 concentration was indicative of complement activation, a two-way ANOVA with Tukey’s post-hoc test was used to assess differences in synovial fluid sC5b-C9 (complement activation product) between patients from the highest and lowest quartiles of synovial fluid C5 levels for both sexes. Results were reported as mean difference +/- 95% confidence intervals.

To assess sex-differences in classical pathway activation, we used an unpaired, two-tailed t-test to measure any differences in percent positive synovial lining cells for C1q in males and females. To identify whether synovial lining C1q was indicative of complement activation, a two-way ANOVA with Tukey’s post-hoc test was used to assess differences in percent positivity of synovial lining cells for C1q between patients from the highest and lowest quartiles of synovial fluid C5 levels for both sexes. Results were reported as mean difference +/- 95% confidence intervals.

#### 2.6.3 Multivariate Linear Regression

A series of multivariate linear regression models were fitted to test the association between median synovial vascular pathology scores (predictor) and synovial fluid C5 concentration (outcome) while adjusting for age, sex, and BMI. To test whether the relationship between C5 and vascular pathology depended on sex, we included sex by C5 concentration interaction terms. As recommended by SAGER guidelines (28), sex-disaggregated analyses were then performed and the estimated marginal means and their respective 95% confidence intervals for C5 concentration and vascularization were reported separately for male and female sex. Unstandardized beta coefficients were reported in the regression model tables to represent change per 1 unit (ng/mL) increase in C5 concentration. To aid interpretation, we also reported the unstandardized beta coefficients per 100 units (ng/mL) increase in C5 concentration (Results text and Figure). Data were linear with normally distributed and homoscedastic residuals. Variance inflation factor (VIF) was used to assess multicollinearity, and all variables had a VIF of < 5.

## 3 Results

A total of 126 patients with late-stage knee OA were screened for eligibility. Four (3.17%) were excluded because they did not have synovial biopsies available. Twenty-four (19.05%) were excluded due to having inadequate synovial fluid volumes (18; 14.29%) or blood contamination (6; 4.76%). One (0.79%) patient was excluded due to low bead counts during the multiplex assay. Therefore, a total of 97 patients with complete synovial fluid biomarker and histopathology data were included in this analysis. Demographics and clinical characteristics for the cohort are presented in **Table 1**. Male and female participants had similar mean and ranges of age, BMI, radiographic knee OA grade, and KOOS pain score.

**Table 1:** Demographic and clinical characteristics of participants (n=97).

	Total Cohort (n=97)	Males (n=52)	Females (n=45)	Difference between Males - Females Mean (95%CI) *p*
**Age, mean ± SD (range)**	67.20 ± 8.53 (44-85)	67.39 ± 8.79 (44-85)	66.98 ± 8.31 (53-82)	0.41 (-3.06, 3.87) *p*= 0.82
**BMI, mean ± SD (range)**	32.60 ± 5.64 (21.10-47.18)	31.93 ± 4.61 (24.20-47.18)	33.38 ± 6.61 (21.10-45.60)	-1.45 (-3.72, 0.83) *p*= 0.21
**KL Grade, frequency (%)**			
3	40 (41.24)	22 (42.31)	18 (40.00)	0.02 (-0.18, 0.22) *p*= 0.84
4	57 (58.76)	30 (57.69)	27 (60.00)	
**KOOS Pain Score, mean ± SD (range)**	47.20 ± 16.42 (0-89)	48.69 ± 14.87 (17-89)	45.47 ± 18.07 (0-83)	3.23 (-3.41, 9.87) *p*= 0.34

BMI, body mass index; CI, confidence interval; KL, Kellgren-Lawrence; KOOS, Knee Injury and Osteoarthritis Outcomes Score; MBL, Mannose-binding lectin; p, p-value; SD, standard deviation.

### 3.1 Synovial Fluid Complement Factors

**Figure 1:**
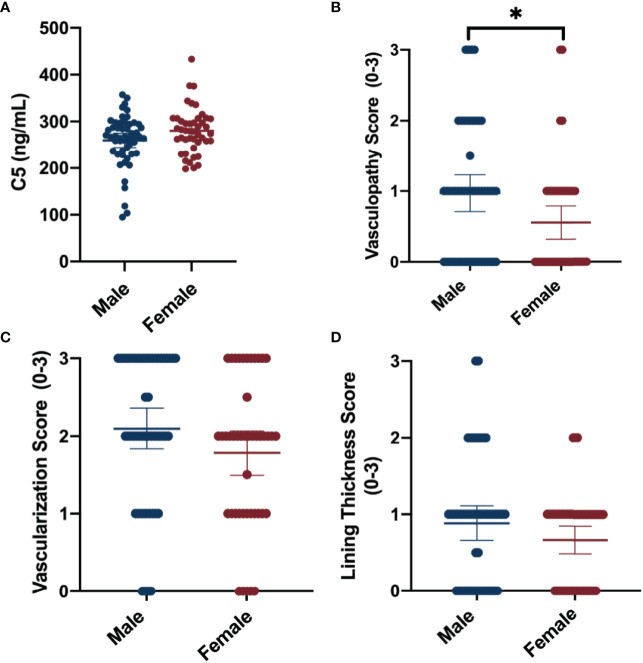
Summary of sex -differences in complement and synovial vascular pathology. Data shown include means ± 95%CI and mean differences between sexes for **(A)** synovial fluid C5 concentrations (ng/mL), **(B)** median vasculopathy score, **(C)** median vascularization score, and **(D)** median synovial lining thickness score. Vascular pathologies were scored on a scale of 0 to 3 (none-severe). *P-value <0.05.

Synovial fluid concentrations of complement factors are reported for the total cohort and separated by sex in **Table 2**. The mean synovial fluid C5 concentration of the cohort was 268.96 ng/mL. Males trended toward lower synovial fluid C5 levels (-20.93 [95%CI -42.08, 0.23]) compared to females (**Table 2**; **Figure 1**). No clear sex differences were found in synovial fluid levels of adipsin (factor D), C2, CFI, or MBL.

**Table 2:** Synovial fluid complement concentrations: total cohort and disaggregated by sex.

	Total Cohort (n= 97) mean ± SD (range)	Males (n= 52) mean ± SD (range)	Females (n= 45) mean ± SD (range)	Difference between Males - Females Mean (95%CI) *p*
Adipsin (ng/mL)	10.67 ± 2.85 (2.72-18.94)	11.06 ± 3.16 (2.72-18.94)	10.22 ± 2.40 (3.59-13.91)	0.83 (-0.31, 1.98) *p*= 0.15
C2 (ng/mL)	311.23 ± 82.47 (112.61-592.12)	317.29 ± 88.33 (112.61-592.12)	304.24 ± 75.51 (131.75-445.15)	13.05 (-20.35, 46.45) *p*= 0.44
C5 (ng/mL)	268.96 ± 53.12 (94.80-432.95)	259.25 ± 55.59 (94.80-357.25)	280.17 ± 48.31 (198.01-432.95)	-20.93 (-42.08, 0.23) *p*= 0.05)
CFI (ng/mL)	73.52 ± 26.35 (8.04-140.03)	73.79 ± 27.84 (8.99-140.03)	73.21 ± 24.83 (8.04-116.70)	0.58 (-10.13, 11.29) *p*= 0.91
MBL* (ng/mL)	28.16 ± 20.28 (1.58-122.70)	28.39 ± 19.81 (1.58-77.01)	27.91 ± 21.03 (4.12-122.70)	-1.84 (-6.96, 7.53) *p*= 0.99

*MBL concentrations were not normally distributed, hence a Mann-Whitney U test measured differences in median MBL concentrations between sexes as opposed to means.

C2, complement component 2; C5, complement component 5; CI, confidence interval; CF1, complement factor 1; MBL, mannose-binding lectin; p, p-value; SD, standard deviation.

### 3.2 Sex-Specific Associations Between C5 and Other Complement Factors in Synovial Fluid

In males, there was no evidence of associations between C5 and other complement factors C2, MBL, CFI, and adipsin (**Table 3**). In females, synovial fluid concentrations of C5 and C2 were positively associated (0.25 [95%CI 0.06 to 0.45]) (**Table 3**). No other complement factors were associated with C5 in females.

**Table 3:** Multivariate linear regression model estimates for C5 concentration with synovial fluid C2, MBL, adipsin, CFI concentration (ng/mL).

	*Adjusted β coefficient (95%CI) *p*
**Male (n= 52)**	
C2	0.11 (-0.07, 0.29) *p*= 0.25
MBL	0.30 (-0.51, 1.11) *p*= 0.46
CFI	-0.39 (-0.96, 0.18) *p*= 0.18
Adipsin	-3.57 (-8.72, 1.58) *p*= 0.17
**Female (n= 45)**	
**C2**	**0.25 (0.06, 0.45) *p*= 0.01**
MBL	0.20 (-0.50, 0.91) *p*= 0.56
CFI	-0.01 (-0.62, 0.60) *p*= 0.96
Adipsin	-0.21 (-6.41, 5.99) *p*= 0.95

*Adjusting for age and BMI.

CI, confidence interval; p, p-value. Regression model estimates with 95% CI excluding 0 are in bold.

### 3.3 Sex-Differences in Synovial Vascular Pathology

The average of the median synovial histopathology scores of the total cohort, as well as the averages separated by sex, are shown in **Table 4**. Males had higher median vasculopathy score (0.42 [95%CI 0.07, 0.77]) compared to females, and demonstrated trends toward higher median vascularization (0.32 [95%CI -0.07, 0.70)] and lining thickness scores (0.22 [95%CI -0.07, 0.51]); however, the latter estimates lacked precision (**Figure 1**). No clear sex differences were detected between sexes for median perivascular edema, infiltrate, fibrosis, or fibrin deposition scores.

**Table 4:** Median synovial vascularization: total cohort and disaggregated by sex.

	Total Cohort (n= 97) mean ± SD (range)	Males (n= 52) mean ± SD (range)	Females (n= 45) mean ± SD (range)	Mean Difference (Males – Females) (n= 97) mean (95%CI) *p*
Median vascularization	1.95 ± 0.96 (0-3)	2.10 ± 0.95 (0-3)	1.78 ± 0.96 (0-3)	0.32 ± 0.19(-0.07, 0.70) *p*= 0.10
Median perivascular edema	0.55 ± 0.65 (0-3)	0.52 ± 0.66 (0-3)	0.58 ± 0.63 (0-3)	-0.06 ± 0.13 (-0.32, 0.20) *p*= 0.66
Median vasculopathy	0.78 ± 0.89 (0-3)	0.97 ± 0.93 (0-3)	0.56 ± 0.78 (0-3)	**0.42 ± 0.18 (0.07, 0.77) *p*= 0.02**
Median lining thickness	0.78 ± 0.72 (0-3)	0.89 ± 0.80 (0-3)	0.67 ± 0.60 (0-3)	0.22 ± 0.15 (-0.07, 0.51) *p*= 0.14
Median infiltrate	1.12 ± 0.85 (0-3)	1.09 ± 0.94 (0-3)	1.16 ± 0.74 (0-3)	-0.07 ± 0.17 (-0.41, 0.28) *p*= 0.69
Median fibrosis	1.24 ± 0.75 (0-3)	1.23 ± 0.70 (0-3)	1.24 ± 0.80 (0-3)	-0.01 ± 0.15 (-0.32, 0.29) *p*= 0.93
Median fibrin deposition	0.87 ± 0.34 (0-1)	0.83 ± 0.38 (0-1)	0.91 ± 0.29 (0-1)	-0.08 ± 0.07 (-0.22, 0.05) *p*= 0.23

CI, confidence interval; p, p-value; SD, standard deviation.

No scores above grade 1 (mild) were assigned for fibrin deposition. Mean sex-differences in histopathology scores with 95% CI excluding 0 are in bold.

### 3.4 Synovial Fluid C5 Concentration is Associated with Synovial Tissue Vascularization in Males

Multivariate linear regression showed that synovial fluid C5 concentrations were inversely associated with median synovial vascularization, while adjusting for age, sex, and BMI (-0.003 [95%CI -0.006, 0.001]; **Supplementary Table 1**); however, the 95% confidence intervals included 0. As recommended by SAGER guidelines and the complement literature, we then assessed potential sex differences by fitting models including a sex by C5 concentration interaction term (**Table 5**) and performed sex-disaggregated analyses (**Table 6**).

**Table 5:** Multivariate linear regression model estimates for the association between vascular pathology and C5 synovial fluid concentration, including sex by C5 concentration interaction terms. (n= 97).

	Adjusted* β coefficient (95%CI) *p*
**Model 1: Vascularization**	
Sex	
Male	Reference
Female	-1.84 (-3.92, 0.23) *p*= 0.08
**C5 concentration**	**-0.005 (-0.010, -0.0001) *p*= 0.04**
Sex × C5 concentration	0.006 (-0.002, 0.014) *p*= 0.12
**Model 2: Vasculopathy**	
Sex	
Male	Reference
Female	-1.69 (-3.63, 0.25) *p*= 0.09
C5 concentration	-0.002 (-0.007, 0.002) *p*= 0.33
Sex × C5 concentration	0.005 (-0.002, 0.012) *p*= 0.19
**Model 3: Perivascular edema**	
Sex	
Male	Reference
Female	0.57 (-0.86, 2.01) *p*= 0.43
C5 concentration	0.003 (-0.001, 0.006) *p*= 0.12
Sex × C5 concentration	-0.002 (-0.007, 0.003) *p*= 0.42

*Adjusting for age and BMI.

Bold values indicate significance at the 5% level.

CI, confidence interval; p, p-value.

**Table 6:** Sex-disaggregated multivariate linear regression model estimates for C5 concentration and vascular pathology.

	*Adjusted β coefficient (95%CI) *p*
**Male (n= 52)**	
**Model 1: Vascularization**	
**C5 concentration**	**-0.005 (-0.009, -0.0001) *p*= 0.04**
**Model 2: Vasculopathy**	
C5 concentration	-0.002 (-0.007, 0.003) *p*= 0.36
**Model 3: Perivascular edema**	
C5 concentration	0.003 (-0.001, 0.006) *p*= 0.13
**Female (n=45)**	
**Model 1: Vascularization**	
C5 concentration	0.002 (-0.004, 0.008) *p*= 0.56
**Model 2: Vasculopathy**	
C5 concentration	0.003 (-0.002, 0.009) *p*= 0.19
**Model 3: Perivascular edema**	
C5 concentration	0.0002 (-0.004, 0.004) *p*= 0.94

*Adjusting for age and BMI.

CI, confidence interval; p, p-value. Regression model estimates with 95% CI excluding 0 are in bold.

**Figure 2:**
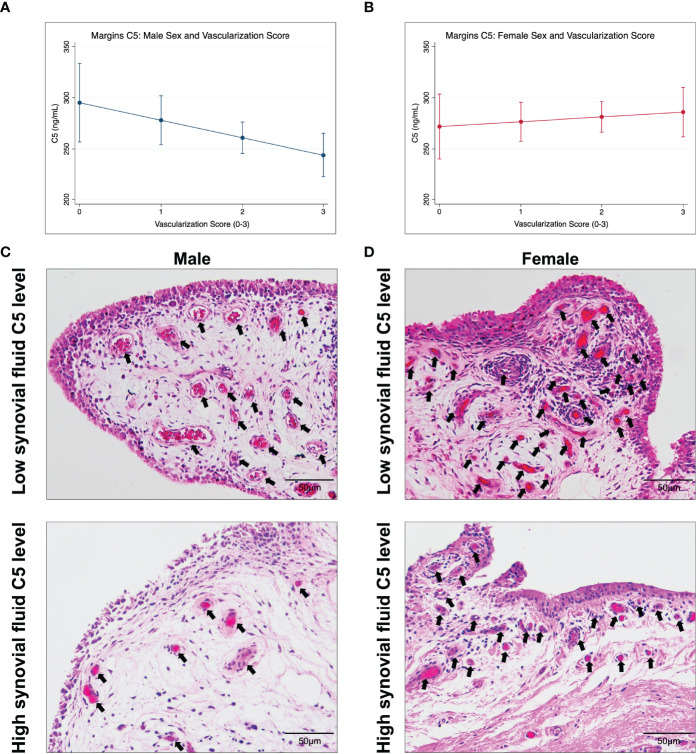
Marginal effects for the association between synovial fluid C5 levels and median synovial vascularization by sex and representative synovial histopathology. Data shown are estimated marginal means ± 95% CIs for median vascularization score (0-3; none-severe) at C5 concentration (ng/mL) of 100, 200, 300, and 400 ng/mL by **(A)** male and **(B)** female sex. Histopathological images of synovium stained with hematoxylin and eosin, representing the C5 and vascularization trend in **(C)** males, and lack of trend in **(D)** females. **(C)** shows synovium from a male with low C5 concentration and high vascularization and from a male with high C5 and less vascularization (arrows). Scale bar 50 µm.

Sex-specific predictive marginal means graphs are shown in **Figure 2** and represent the association between synovial fluid C5 concentration (at 100 ng/mL intervals) and median vascularization scores (at 1-unit intervals). Sex-disaggregated analyses suggest that for every 100ng/mL increase in C5 concentration, there was a 0.5-unit decrease (beta = -0.005 [95%CI, -0.009, -0.0001] per 1 ng/mL change) in median vascularization score in males (**Table 6** and **Figure 2A**). However, there was no evidence of a relationship between C5 concentration and vascularization identified in females (**Figure 2B**). In males but not females, similar but weaker trends of association were seen between synovial fluid C5 levels and increased synovial tissue perivascular edema and decreased vasculopathy in males; however, the 95%CIs were imprecise (**Table 6**). Representative synovial histopathology images for both sexes from the highest/lowest synovial fluid C5 quartile groups are shown in **Figure 2C**; lower vascularization was observed in males in the highest C5 quartile, but similar vascularization between highest and lowest C5 quartiles in females.

### 3.5 Sex-Differences in Synovial Fluid Complement Activation

**Figure 3:**
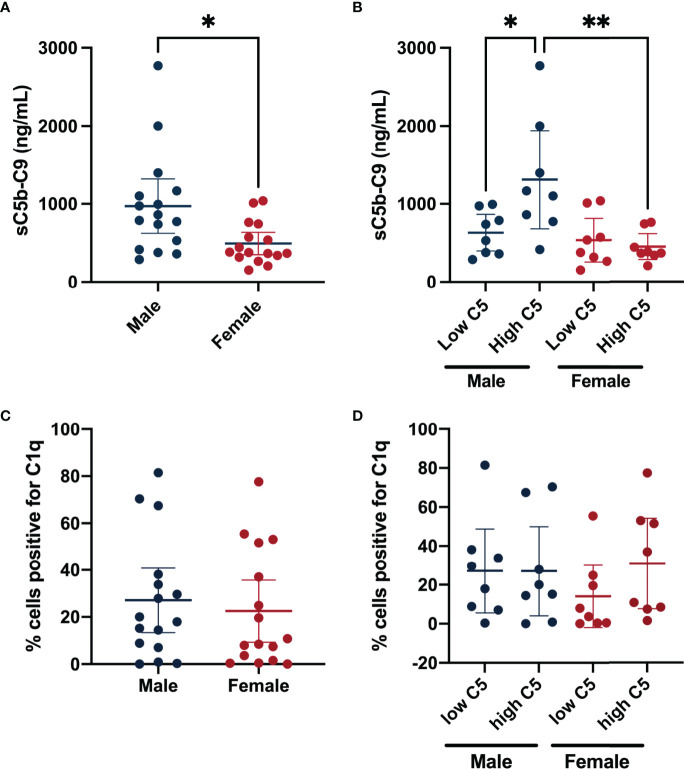
Synovial fluid terminal complement complex sC5b-C9 levels and synovial lining C1q grouped by sex and synovial fluid C5 quartile. Data shown are mean sC5b-C9 concentrations (ng/mL) ± 95% CIs for **(A)** Male (n=16) and Female (n=16). **(B)** Represents sub-groups of males with low synovial fluid C5 concentrations (n= 8), males with high synovial fluid C5 concentrations (n= 8), females with low synovial fluid C5 concentrations (n= 8), and females with high synovial fluid C5 concentrations (n= 8). **(C)** Shows mean percent positive synovial lining cells for C1q ± 95% CIs for males (n=16) and females (n=16). **(D)** Shows the percent positive synovial lining cells for C1q of patients from the highest and lowest quartiles of synovial fluid C5 levels for both sexes. *P-value <0.05; **P-value <0.005 for between-group comparisons of synovial fluid sC5b-C9 concentrations.

We compared sC5b-9 levels in synovial fluid between subgroups of male and female patients in the highest and lowest synovial fluid C5 quartiles. Overall, males had higher synovial fluid sC5b-C9 levels (477.70 [95%CI 118.10, 837.30]) compared to females (**Figure 3A**). Similarly, comparing males and females from the highest synovial fluid C5 quartiles also demonstrated higher sC5b-C9 levels in males (858.5 ng/mL [95%CI 249.60, 1467.00]) than females (**Figure 3B**). When disaggregated by sex, synovial fluid sC5b-C9 concentrations were higher in males with high synovial fluid C5 (678.6 ng/mL [95%CI 69.73, 1287.00]) compared to males with low synovial fluid C5 concentrations (**Figure 3B**). In contrast, similar levels of synovial fluid sC5b-C9 were measured in females regardless of synovial fluid C5 levels.

To determine if classical complement pathway activation might differ between sexes, we measured C1q deposition in synovial lining. However, no differences between sexes, regardless of synovial fluid C5 level, were identified (**Figures 3C, D**).

## 4 Discussion

Complement factor expression in joint tissues is associated with synovitis (18, 29), worse pain (30), and disease progression (27, 31). Genetic and pharmacologic inhibition of complement in mouse models of OA have shown protective effects against structural joint damage (18). Although complement activation is associated with vascular pathology in diseases such as retinopathy (32–34), no such associations have been determined for OA. Further, well-recognized sex-differences exist in complement activation (23) and OA outcomes (21, 35). In this study, we investigated whether complement levels are related to synovial vascular pathology, and whether sex-differences may interact with this relationship. We focused on late-stage knee OA where vascular pathology is likely greatest and matched synovial fluids and tissues are readily available. Our primary analysis included C5 because it is the first factor in the terminal effector pathway and C5 deletion protects against OA development in experimental mouse models (18), suggesting that higher C5 levels may be associated with worse OA outcomes. We found that males with higher total C5 complement have reduced synovial vascularization, but this association does not occur in females. Interestingly, males with higher synovial fluid C5 had increased terminal complement activation in synovial fluid, whereas higher C5 levels did not correspond to higher terminal complement activation in females. These findings raise some interesting questions that require further investigation.

Total C5 complement was inversely associated with vascularization, raising the hypothesis that complement signaling might inhibit synovial vascularization in OA. However, due to our cross-sectional design, we cannot confirm any causal mechanistic relationship. Indeed, the null hypothesis would indicate that the association is an epiphenomenon where pathophysiological OA conditions that lead to greater C5 levels and terminal complement activation in males also have an inhibitory effect on synovial vascularization through a mechanism independent of complement. Notwithstanding, current knowledge from other diseases suggests that an epiphenomenon is less likely, since complement signaling regulates pathological angiogenesis in multiple diseases involving chronic inflammation. Our work therefore indicates that prospective interventional studies should be completed to test potential inhibitory effects of C5 activation on vascular pathology. If complement inhibits synovial vascularization, this has important implications for joint healing in knee OA. In particular, the synovium is critical to restore and maintain joint homeostasis and impairment of normal wound healing processes by interfering with synovial angiogenesis is one potential mechanism through which complement may lead to worse knee OA outcomes.

Previous studies have suggested that complement may be activated by numerous proteases secreted into the OA joint environment (18). Since multiple complement pathways converge on C5 to generate C5a and C5b activation fragments, which in turn activate C5a receptors and the terminal effector pathway (sC5b-9) respectively, synovial fluid total C5 levels therefore serve as a useful proxy indicator of the potential for complement activation in OA joints. Accordingly, we focused our primary analyses on C5 levels in synovial fluid and confirmed that higher levels of C5 in males are associated with higher sC5b-9, indicating greater complement activation. Further, continuous physiologic turnover of parent complement factors such as C5 *in vivo* leads to the generation of activation fragments that are proportional to the level of the parent factor in healthy and disease states. However, in some diseases such as in systemic lupus erythematosus, very rapid activation may exceed the rate of synthesis leading to consumption of the parent factor. Even though excessive complement activation to the point of consumption is very unlikely in OA, we conducted secondary analyses in sub-groups of patients with high or low total C5 levels and confirmed this is not the case. These secondary analyses were therefore critical in allowing us to determine that the inverse relationship between total C5 levels and synovial vascularization suggests the hypothesis that an anti-angiogenic role for complement may exist in males with knee OA, rather than a pro-angiogenic role.

Numerous studies have reported pro- or anti-angiogenic roles of complement signaling. Importantly, disease context and the pathological cell types involved may influence the divergent roles of complement (11). If we interpret our findings using the concept that OA resembles a chronic wound as proposed by others (9), OA-related angiogenesis may be driven by pro-angiogenic wound healing mechanisms that go awry due to innate-driven chronic inflammation interrupting normal healing (36). Indeed, some complement factors such as C1q may promote canonical angiogenesis in healthy and wounded tissue (37), whereas components of the C5-C5a axis may exert pro-angiogenic effects in healthy contexts but disrupt angiogenic processes in chronic wounds. Considering our findings of an inverse association between C5 complement and vascularization in males with OA, innate inflammation mechanisms including complement may interfere with wound healing-related angiogenesis. For example, in a model of experimental wound healing, mice deficient in C3, C5, and C5aR1 had better cutaneous wound healing compared to wild-type mice (38). This study also found that C5a-mediated immune cell activation delayed wound healing and angiogenesis (11, 38). Further, in a surgical model of post-traumatic knee OA, mice with C5-deficiency had less cartilage degradation and had lower expression of pro-inflammatory mediators compared to mice with normal C5 (18), although synovial vascularization was not assessed. Notably, all of these studies were completed using male mice. Considering our data in context with these findings, pathological complement activation may lead to increased inflammation and derangements in angiogenesis, with important implications in OA pathogenesis and synovial vascular pathology. Although complement activation can influence inflammatory processes such as immune cell recruitment, activation, metabolism, apoptosis, and interactions with the adaptive immune system (39), we did not identify any relationships between synovial fluid complement levels and other inflammatory features of synovial histopathology (*e.g.*, lining thickening, immune infiltrate, fibrin deposition). However, we cannot rule out complement-related changes in inflammatory mediators such as cytokines or inflammatory cell function, as these were not measured in this study. Future studies investigating how complement activation directly affects synovial vascular pathology and how this may impact clinical outcomes in knee OA patients is warranted, and such studies should investigate sex differences.

**Figure 4:**
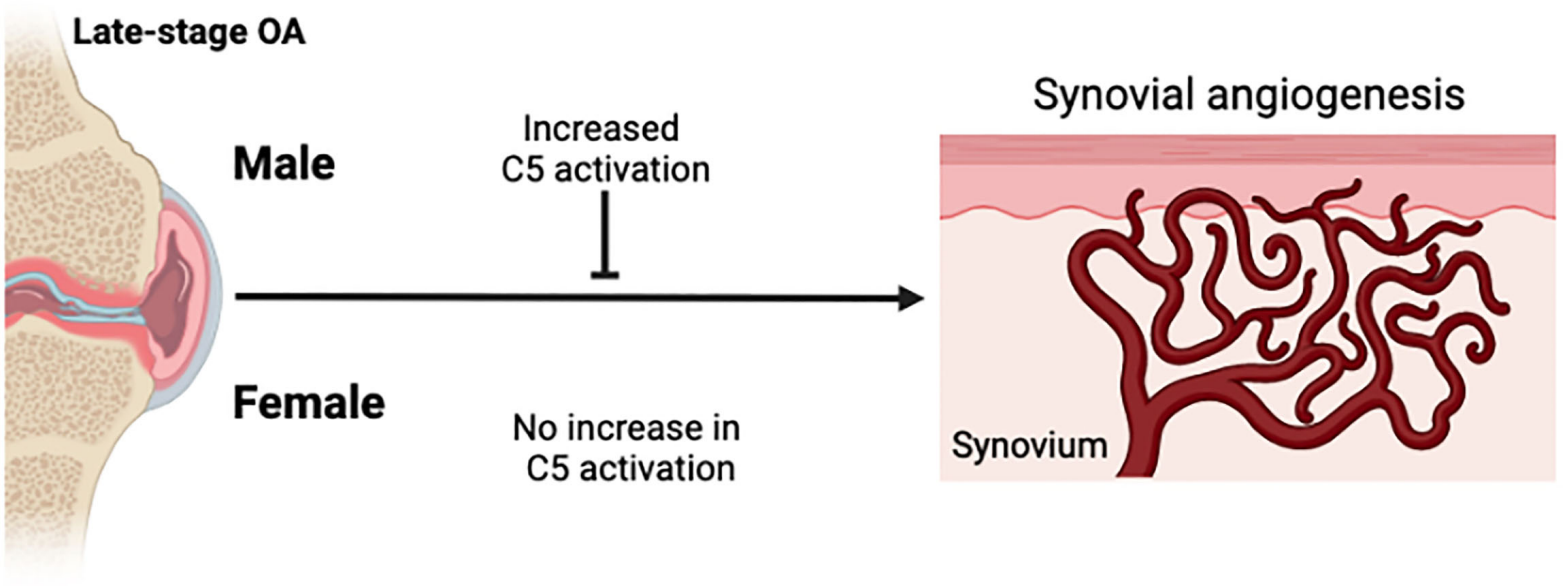
Schematic summary of sex-differences in complement activation and synovial angiogenesis. Knee OA is associated with synovial tissue angiogenesis. In males with knee OA, complement activation was increased and associated with reduced synovial vascularization. In females with knee OA, complement activation was not increased and no association between complement activation and synovial vascularization was detected. Figure created in Biorender.com.

Our sex-specific analyses suggest that complement activation may play more of a role in synovial vascular pathology mechanisms in males than it does in females with knee OA (**Figure 4**). Males with higher C5 levels had higher levels of complement activation (sC5b-C9) compared to males with lower C5 levels and compared to females with high C5 levels. This suggested that females with knee OA do not activate terminal complement as effectively as males do, regardless of the level of C5 present in synovial fluid. Sex-specific regulation of synovial fluid complement activation at the level of C5 or subsequent steps in the cascade may be important in knee OA pathophysiology. Females with knee OA appear to have a reduced ability to activate complement and/or convert C5b intermediate to sC5b-C9 in synovial fluid. This finding corroborates previous studies demonstrating lower levels of terminal pathway components in the serum of healthy females compared to males (23), which has also been shown in mice (40) but has not previously been demonstrated in synovial fluid. Several candidate mechanisms may mediate sex differences in how complement influences disease processes, which should be explored in future studies. For example, CD59 may be expressed at higher levels in the synovium of female patients, which could prevent the conversion of the MAC complex after cleavage of C5 (41). Sex differences in the inflammatory cell composition and/or function of the synovium might differentially regulate complement activation between sexes or responses to complement signaling. Gaya da Costa et al. also showed that serum levels of C3 and properdin were lower in females compared to males, while the complement activator Factor D (adipsin) levels were significantly higher (23). Although we did not measure C3 levels in this study, we did not find any sex-differences in synovial fluid levels of adipsin, other complement factors, nor the complement inhibitor CFI, in patients with late-stage knee OA. The differences between our study and others are likely due to differences between complement expression in serum vs. synovial fluid since complement factor production by local joint tissues is dysregulated in OA; other differences in cohort demographic factors including age, BMI, or ethnicity may have also played a role. It is also notable that most murine studies are done using male mice alone, without comparative analyses in females, although this trend is starting to improve in the OA research field in recent years. Considering our findings that complement activation was not associated with vascularization in females, comparative analyses in both sexes remains an important gap in the literature and should be considered carefully in future studies, especially those examining complement roles in disease.

Upstream complement pathway activation was not assessed in this study but may influence downstream events. Synovial fluid levels of C5 trended to be higher in females than in males and were associated with C2 levels in females only. This suggested a potential sex-specific link between C5 levels and classical pathway activation in females, but we found no sex differences in C1q deposition in synovial tissues regardless of synovial fluid C5 levels. However, we cannot rule out a link between C1q and synovial vascularization since C1q has also been linked with angiogenesis through mechanisms that bypass downstream complement activation (37), and our analysis was limited to subgroups of patients based on total C5 level. In addition to playing a role in classical pathway activation (23, 42), mixed reports also suggest a role for C2 in lectin pathway activity. Gaya da Costa et al. did not find any association between C2 concentrations and lectin pathway activity in serum of healthy individuals (24), while a previous study showed that human serum deficient in C2 ablated lectin pathway activity (42). Although our study was focused on downstream effector pathway associations, upstream pathways should be investigated for sex differences in different disease contexts.

Our study is limited to cross-sectional relationships. Although we cannot conclude that causal relationships exist between complement and synovial vascular pathology, our findings raise the novel hypothesis that complement signaling inhibits synovial angiogenesis in knee OA and that sex differences modify these effects. The focus on late-stage knee OA also limits the generalizability of our findings to the context of early knee OA. Future studies should also assess longitudinal relationships since increased vascularization has been shown to be associated with increased KL grade at follow up (7). Nearly 15% of patients were excluded due to insufficient synovial fluid volumes at time of aspiration, which may have influenced our findings, although it remains uncertain whether this would lead to over-estimation, under-estimation, or no effect. Although our sample size was met for the primary analysis, larger samples are needed (*e.g.*, 70-80 per group) to be appropriately powered for the sex-disaggregated analyses. Synovial angiogenesis is a pathological feature in knee OA and associated with worse joint pain yet, it is not known whether disease-related or therapeutic reduction of synovial vascularization would be protective or lead to worse disease outcomes. Given the strong relationship between synovial vascularization and OA symptoms, the effects of reducing synovial angiogenesis on OA outcomes should be investigated.

This is the first study to describe an association between synovial fluid complement C5 levels and synovial tissue microvascular pathology in patients with knee OA. Importantly, we also identified sex differences in this association and in synovial fluid complement activation. Higher synovial fluid C5 levels were associated with greater terminal complement activation and decreased synovial vascularization in males, but neither occurred in female patients. These findings raise the hypothesis that sex differences in synovial fluid complement activation in knee OA may lead to differential effects on synovial microvascular pathology. Prospective studies are required to confirm whether complement directly or indirectly inhibits synovial angiogenesis or is merely an indicator of the inflammatory milieu within the joint. Our findings also serve as a compelling reminder to include sex-disaggregated analyses in OA research.

## Data Availability Statement

The original contributions presented in the study are included in the article/**Supplementary Material**. Further inquiries can be directed to the corresponding author.

## Ethics Statement

The studies involving human participants were reviewed and approved by Western University Health Sciences Research Ethics Board. The patients/participants provided their written informed consent to participate in this study.

## Author Contributions

ES and HP contributed to study design, data collection and analysis, and drafted the manuscript. MC contributed to data analysis. TB and TA contributed to study design, data interpretation and critically revised the manuscript. All authors read and approved final manuscript.

## Funding

This work was funded in part by grants from the Academic Medical Organization of Southwestern Ontario (AMOSO; INN17-004), the Canada Research Chairs program, and Western University’s Bone and Joint Institute. HP is supported by Frederick Banting and Charles Best Doctoral Award from CIHR.

## Conflict of Interest

The authors declare that the research was conducted in the absence of any commercial or financial relationships that could be construed as a potential conflict of interest.

## Publisher’s Note

All claims expressed in this article are solely those of the authors and do not necessarily represent those of their affiliated organizations, or those of the publisher, the editors and the reviewers. Any product that may be evaluated in this article, or claim that may be made by its manufacturer, is not guaranteed or endorsed by the publisher.
