# Supplementary material for: Sex-Differences and Associations Between Complement Activation and Synovial Vascularization in Patients with Late-Stage Knee Osteoarthritis
Source: Front Immunol. 2022 May 24;13:890094. doi: 10.3389/fimmu.2022.890094 (PMC9170895; doi:10.3389/fimmu.2022.890094)
Supplement: Supplementary file 1 [file Table_1.docx]

Supplementary Material

| **Supplementary Table 1. Multivariate linear regression model estimates for C5 concentration and vascular pathology in full cohort (n=97)** | | | |
| --- | --- | --- | --- |
|  | **β coefficient** | **SE** | **95% CI (p-value)** |
| **Model 1: Vascularization** |  |  |  |
| C5 concentration (ng/mL) | -0.003 | 0.002 | -0.006 to 0.001 (p=0.19) |
| **Model 2: Vasculopathy** |  |  |  |
| C5 concentration (ng/mL) | -0.0003 | 0.002 | -0.004 to 0.003 (p=0.85) |
| **Model 3: Perivascular edema** |  |  |  |
| C5 concentration (ng/mL) | 0.002 | 0.001 | -0.001 to 0.004 (p=0.18) |
| Adjusting for age, sex, and BMI  CI, confidence interval; SE, standard error | | | |
